# Draft Genome Sequence of *Vibrio splendidus* DSM 19640

**DOI:** 10.1128/genomeA.01368-17

**Published:** 2017-11-30

**Authors:** Cynthia Maria Chibani, Anja Poehlein, Olivia Roth, Heiko Liesegang, Carolin Charlotte Wendling

**Affiliations:** a Department of Genomic and Applied Microbiology and Göttingen Genomics Laboratory, Institute of Microbiology and Genetics, University of Goettingen, Göttingen, Germany; b GEOMAR Helmholtz Centre for Ocean Research, Evolutionary Ecology of Marine Fishes, Kiel, Germany

## Abstract

Here, we present the draft genome sequence of *Vibrio splendidus* type strain DSM 19640. *V. splendidus* is an abundant species among coastal vibrioplankton. The assembly resulted in a 5,729,362-bp draft genome with 5,032 protein-coding sequences, 6 rRNAs, and 117 tRNAs.

## GENOME ANNOUNCEMENT

*Vibrio splendidus*, an opportunistic marine pathogen, is a dominant member of the costal vibrioplankton and one of the causative agents of vibriosis (1). Vibriosis affects marine wildlife, as well as aquaculture, and thus leads to major economic losses (2). *V. splendidus* can cause high mortality rates in larval and juvenile marine animals, including turbots, oysters, clams, and scallops (3, 4) and can even spread to humans through the consumption of infected seafood.

The pathogenicity of *V. splendidus* is multifactorial and regulated by intrinsic and extrinsic factors. While increasing water temperatures favor its transmission and proliferation, an extracellular metalloprotease (Vsm) has been shown to be the major determinant of toxicity in Pacific oysters (5). Despite a significant body of empirical research, our understanding of the factors that contribute to the virulence of *V. splendidus* is far from being complete.

Genomic DNA of *V. splendidus* DSM 19640 was extracted with the MasterPure complete DNA purification kit (Epicentre, Madison, WI, USA) and used to generate Illumina shotgun paired-end sequencing libraries. Sequencing was performed employing the MiSeq system and the MiSeq version 3 reagent kit (600 cycles), as recommended by the manufacturer (Illumina, San Diego, CA, USA). Quality filtering using Trimmomatic version 0.32 (6) resulted in 2,483,315 paired-end reads. *De novo* genome assembly was performed with the SPAdes version 3.11.0 genome assembler (7). The assembly resulted in 66 contigs (>500 bp) and an average coverage of 18,876-fold. The assembly was validated, and the read coverage was determined with QualiMap version 2.1 (8).

The draft genome of *V. splendidus* DSM 19640 consisted of 5,729,362 bp with an overall GC content of 43.91%. Genome annotation was performed using Prokka (9). In total, 6 rRNAs, 117 tRNAs, 3,416 protein-coding sequences with predicted functions and 1,616 genes with unknown functions were determined.

A search for toxins in the genome revealed the following three putative virulence factors: (i) a gene encoding a protein identical to the *V. cholerae* zona occludens toxin (VSPL_25760), (ii) a protein identical to the transmembrane regulatory protein ToxS involved in the regulation of *V. cholerae* toxins, and (iii) a protein with 93.65% identity to the metalloprotease Vsm known from *V. splendidus* strain JZ6 (VSPL_47630).

This is the first report of the genome sequence of *V. splendidus*. It will provide further insights into the virulence mechanisms of this pathogen and will also serve as a platform to facilitate comparative genomics of other *Vibrio* species.

### Accession number(s).

This whole-genome shotgun project has been deposited at DDBJ/ENA/GenBank under the accession number PDMZ00000000. The version reported here is the first version, PDMZ01000000.
